# Selective Inner Hair Cell Loss in a Neonate Harbor Seal (*Phoca vitulina*)

**DOI:** 10.3390/ani12020180

**Published:** 2022-01-12

**Authors:** Maria Morell, Laura Rojas, Martin Haulena, Björn Busse, Ursula Siebert, Robert E. Shadwick, Stephen A. Raverty

**Affiliations:** 1 Institute for Terrestrial and Aquatic Wildlife Research, University of Veterinary Medicine Hannover, Foundation, 25761 Büsum, Germany; ursula.siebert@tiho-hannover.de; 2 Zoology Department, The University of British Columbia, Vancouver, BC V6T 1Z4, Canada; shadwick@zoology.ubc.ca; 3 Faculty of Veterinary Medicine and Zootechnics, National Autonomous University of Mexico, Av. Universidad 3000, Delegación Coyoacán, Mexico City 04510, Mexico; laura.fmvz@gmail.com; 4 Vancouver Aquarium Marine Science Center, Vancouver, BC V6G 3E2, Canada; martin.haulena@ocean.org; 5 Department of Osteology and Biomechanics, University Medical Center Hamburg-Eppendorf, 22529 Hamburg, Germany; b.busse@uke.de; 6 Animal Health Center, Ministry of Agriculture, Abbotsford, BC V3G 2M3, Canada; stephen.raverty@gov.bc.ca

**Keywords:** congenital hearing loss, organ of Corti, marine mammals, pinnipeds, scanning electron microscopy, hair cell loss

## Abstract

**Simple Summary:**

Congenital hearing loss (i.e., hearing impairment present at birth) is recognized in humans and other terrestrial species, but there is a lack of information on congenital malformations and associated hearing loss in pinnipeds (seals, sea lions, and walruses). Baseline knowledge on marine mammal inner ear malformations is essential to differentiate between congenital and acquired abnormalities, which may be caused by infectious agents, age, or anthropogenic interactions, such as noise exposure. Analysis of the cochlea of a neonate harbor seal (*Phoca vitulina*) revealed bilateral loss of inner hair cells (sensory cells responsible for transducing the auditory signal) while the outer hair cells (sensory cells responsible for sound amplification and frequency selectivity and sensitivity) were intact. The selective inner hair cell loss (up to 84.6% of loss) was more severe in the basal turn, where the high frequencies are encoded. Potential causes and consequences are discussed. This is the first report of a case of selective inner hair cell loss in a marine mammal neonate, likely congenital.

**Abstract:**

Congenital hearing loss is recognized in humans and other terrestrial species. However, there is a lack of information on its prevalence or pathophysiology in pinnipeds. It is important to have baseline knowledge on marine mammal malformations in the inner ear, to differentiate between congenital and acquired abnormalities, which may be caused by infectious pathogens, age, or anthropogenic interactions, such as noise exposure. Ultrastructural evaluation of the cochlea of a neonate harbor seal (*Phoca vitulina*) by scanning electron microscopy revealed bilateral loss of inner hair cells with intact outer hair cells. The selective inner hair cell loss was more severe in the basal turn, where high-frequency sounds are encoded. The loss of inner hair cells started around 40% away from the apex or tip of the spiral, reaching a maximum loss of 84.6% of hair cells at 80–85% of the length from the apex. Potential etiologies and consequences are discussed. This is believed to be the first case report of selective inner hair cell loss in a marine mammal neonate, likely congenital.

## 1. Introduction

Profound congenital hearing loss (i.e., hearing impairment present at birth) is present in 1–3 children out of 1000 [1,2]. Around 50 to 60% of cases of congenital hearing loss are due to a genetic etiology, while the remainder may be attributed to environmental factors, including noise exposure, ototoxic drug exposure, and protozoal, bacterial, or viral infections [3,4]. Genetic mechanisms of congenital hearing loss are divided into syndromic (when hearing loss occurs along with a variety of other malformations) or non-syndromic (when hearing loss is the only apparent abnormality, which accounts for approximately 70% of cases of genetic-related hearing loss) [5,6]. In humans, half of all the non-genetic causes of congenital hearing loss are attributed to infectious pathogens, including *Toxoplasma gondii*, rubella, cytomegalovirus, herpes, and syphilis infections. Within these infectious agents, congenital cytomegalovirus is the most common cause of non-hereditary sensorineural hearing loss in childhood [2].

The organ of Corti (hearing organ) in mammals is formed by sensory cells that are typically arranged in one row of inner hair cells (IHCs) and three parallel rows of outer hair cells (OHCs). While OHCs amplify the incoming signal and are responsible for frequency sensitivity and selectivity, IHCs transduce the mechanical sound stimulation into the release of neurotransmitters onto the afferent auditory nerve fibers that conduct the auditory information to the brainstem. In mammals, low frequencies are encoded in the apex (apical region or tip of the spiral), and the high frequencies are encoded in the base of the cochlea, closer to the stapes.

Structural alterations can occur as a consequence of severe noise exposure, including loss of entire hair cells, alterations in stereocilia, nuclei karyorrhexis and karyopycknosis, and degeneration of type I innervation, among others [7,8]. Following cochlear hair cell apoptosis, neighboring supporting cells initiate the elimination of the hair cell, leaving a distinct “scar.” This scarring process results in the simultaneous expansion of the supporting cells and sealing of the reticular lamina [9]. The presence of scars among hair cell rows is an important criterion that can be used to assess a possible history of noise-induced hearing loss [10]. However, potential lesions due to noise exposure and other environmental factors in stranded marine mammals can be confused with hair cell loss due to congenital malformations.

Therefore, it is imperative to develop baseline information on the pathogenesis and prevalence of congenital hearing loss in marine mammals, to further differentiate among congenital or acquired lesions, such as infectious pathogens or anthropogenic interactions associated with noise overexposure. Clinical and pathologic examination of neonates provides the optimal information on congenital hearing loss since it is less likely that they have been exposed to any agent that might cause hair cell damage after birth.

Congenital diseases previously reported in harbor seals include cleft palate, cleft lips, cardiac defects, hydronephrosis, hiatal hernia, scoliosis, arthrogryposis, lens triplication, macroglossia, anorectal malformation and vaginal artesia, brain or cranial malformations, dwarfism, intestinal atresia, and neuroglial heterotopia [11,12,13,14,15,16,17]. However, no descriptions of inner ear congenital malformations in harbor seals or other marine mammals have been documented. Herein, we present the index case of a harbor seal neonate with inner ear lesions.

## 2. Materials and Methods

In British Columbia, distressed or abandoned harbor seal pups are reported to the Vancouver Aquarium Marine Mammal Rescue (MMR) or British Columbia Marine Mammal Stranding Response Network as part of the general protocol. Depending on the location and resources, trained volunteers and experienced staff are mobilized to recover animals and transport them for rehabilitation at the MMR. On arrival, animals are triaged and clinically assessed by either an Animal Health Technologist or Veterinarian with experience in marine mammal health. After an initial evaluation, animals are placed individually in large totes and maintained on a milk formula herring-based diet for 3–4 weeks, then weaned. The enclosures are cleaned, and the animals are clinically assessed daily.

On 15 July 2014, a male harbor seal (PV 1475) was admitted to MMR with a history of possible maternal loss or abandonment. The animal was assessed and stabilized. On August 9, the pup was observed to be quiet with a retracted third eyelid, and congested ulcers were noted in the oral cavity. Lip smacking was observed, and an antiemetic was administered. The condition of this animal deteriorated, and on August 10, bloody diarrhea with bloody nasal discharge and dyspnea with open mouth breathing were observed. The animal did not improve after a course of Dexamethasone 5 [5 mg/mL, manufactured by Vetoquinol (Lavaltrie, QC, Canada), dosage administered 0.2 mg/kg], Ceftriaxone Sodium [100 mg/mL reconstituted, manufactured by Sandoz (Boucherville, QC, Canada), dosage administered 20 mg/kg], ceftiofur crystalline free acid Excede [200 mg/mL, manufactured by Zoetis (Kirkland, QC, Canada), dosage administered 7 mg/kg] and benzylpenicillin procaine and benzylpenicillin benzathine suspension Duplocillin LA [dosage administered 1.0 mL of penicillin solution by intramuscular injection, i.e., 150,000 iu of each/mL, manufactured by Merck (Kirkland, QC, Canada)]. Due to a poor prognosis, the pup was humanely euthanized and presented for necropsy. The animal was approximately 1 month of age when it died, with a weight of 9.0 kg and a total length of 58 cm.

An extensive post-mortem examination was conducted following international protocols [18,19] at the Animal Health Center, Abbotsford, British Columbia.

### 2.1. Inner Ear Analysis

**Figure 1:**
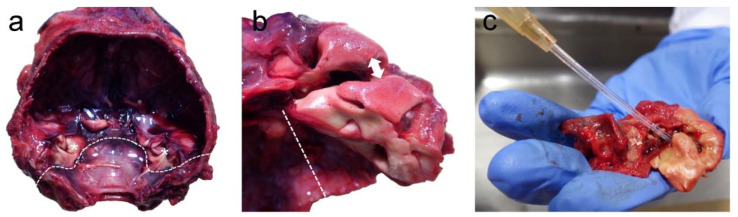
(**a**) Skull after the extraction of the brain with the location of the ears (asterisks). The dotted line indicates the position of the occipitomastoid suture, where the chisel is placed to remove the occipital bone. (**b**) Separation of the periotic from the tympanic bone by first placing the chisel in the location highlighted with the double arrow, and collection of the periotic bone by sectioning the squamosal bone through the dotted line. (**c**) The final step of the perilymphatic perfusion through the oval window with fixative, after extracting the stapes and perforating the round and oval window membranes with a small needle.

The head was removed, and the inner ears were collected at the University of British Columbia (UBC) within 4.25 h post-mortem. The skull was opened with a hand saw to extract the brain, and the occipital bone was removed with a chisel T-Shape (Virchow skull breaker) post-mortem from the occipitomastoid suture (Figure 1a). The ear bones (periotic and tympanic) were separated and extracted from the squamosal bone using a chisel T-shape (Figure 1b), and the inner ears were perfused perilymphatically with 2.5% glutaraldehyde in 0.1M cacodylate buffer (Figure 1c), changed the media into 0.1M cacodylate buffer the following day, and subsequently processed for ultrastructural evaluation, following a previously optimized protocol for marine mammals [10,20,21,22].

### 2.2. Scanning Electron Microscopy (SEM)

The periotic bones (surrounding the cochlea) were decalcified with 14% Ethylenediaminetetraacetic acid (EDTA) tetrasodium salt, changing the media every 7–15 days for 47 and 192 days (right and left ears, respectively). Both cochleae were dissected to remove the bone, vestibular wall, Reissner and tectorial membranes, and dehydrated with increasing concentrations of ethanol.

The right cochlea was critical point dried (Supercritical Autosamdri 815B, Tousimis), coated with platinum/palladium, and imaged with a Hitachi S-4700 SEM at the UBC Bioimaging Facility, Canada. The left cochlea was critical point dried (Bal-Tec CPD030), coated with gold, and imaged with a Zeiss Crossbeam 340 FIB-SEM at the University Medical Center Hamburg-Eppendorf, Germany. The brightness and contrast of images were adjusted in Adobe (San Jose, CA, USA) Photoshop^®^ 2021.

### 2.3. Characterization of the Lesions

The cochlear length was measured with ImageJ^®^ software (https://imagej.nih.gov/ij/index.html accessed on 19 April 2021) from SEM micrographs at the level of the limit between the first row of OHCs and the inner pillar cells. A total of 33 micrographs were used for the calculation of the cochlear spiral from the left ear. Specific points on each image were identified to achieve an accurate consecutive delineation of length. Contiguous measurements avoided overlapping or gaps in the calculation of the structure (see Girdlestone and colleagues [23]).

Once the cochlear length was measured, it was possible to identify equidistant locations every 5% along the cochlear spiral. Due to damage in the end of the base or hook region, the locations were evaluated up to 88.6% from the apex. Counting of IHCs was performed at 5% increments to determine the number of IHCs present and absent. In addition, to confirm the number of absent IHCs at each location, the length of the cuticular plate of the IHCs was measured and averaged every 10% of the cochlear spiral. To illustrate the results, location 5% represents the counting of IHCs from 0 to 5%, location 10% from 5.01 to 10%, onwards.

## 3. Results

### 3.1. Post-Mortem Examination

The animal presented for necropsy in moderate body and good post mortem condition. Morphologic diagnoses included marked bronchopneumonia, with transmural vasculitis and atelectasis, multifocal necrotizing adrenocortical adenitis with intralesional inclusions consistent with phocid herpesvirus infection, hepatocellular hemosiderosis, splenic extramedullary hematopoiesis, and renal congestion. No bacteria were recovered from the lung, but light *Pseudomonas aeruginosa* was cultured from the spleen, with moderate mixed growth of *Staphylococcus* sp., *Corynebacterium* sp., *Psychrobacter* sp., *Escherichia coli,* and *Enterococcus* sp. isolated from a lymph node. Based on the nature of the bacterial isolates and histopathology (vasculitis and pneumonia), the *Pseudomonas aeruginosa* was considered significant. The lack of more significant growth from the lung was attributed to antemortem antimicrobial administration. Molecular studies of pooled tissues (striated muscle, diaphragm, heart, and liver) [24] did not detect Apicomplexa, including *T. gondii*.

### 3.2. Inner Ear Analysis

**Figure 2:**
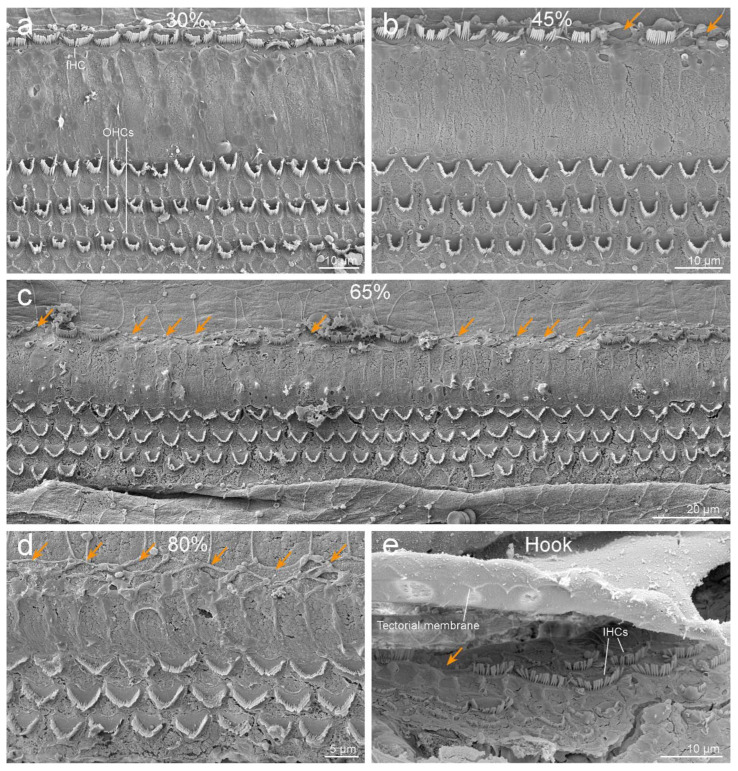
Scanning electron microscopy images of the organ of Corti of the left ear along the cochlear spiral, at 30% (**a**), 45% (**b**), 65% (**c**), and 80% (**d**) distances from the apex. Note that while the outer hair cells (OHCs) are present forming three (and sometimes four) rows, there is a loss of inner hair cells (IHCs, highlighted with orange arrows), with increasing severity towards the base. (**e**) First IHCs of the hook. The three first IHCs are arranged in two rows. The undersurface of the tectorial membrane shows the imprints where the stereocilia of OHCs are inserted.

Ultrastructural evaluation of the organ of Corti revealed selective IHC loss throughout the cochlear spiral (Figure 2). While the OHCs were present forming three and often scattered four rows (Figure 2c), there was a loss of IHCs, which was more severe towards the base of the cochlea. The loss of IHCs was determined by the detection of scars, resulting from the overgrowth of adjoining, supporting cells (orange arrows in Figure 2).

**Figure 3:**
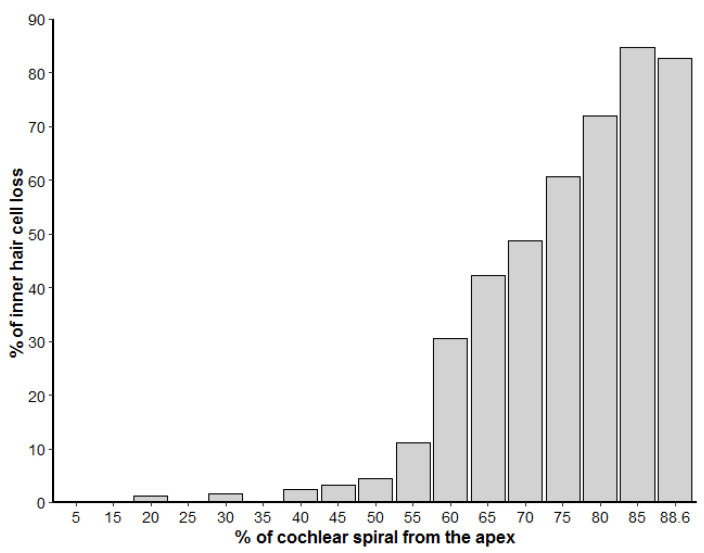
Loss of inner hair cells along the left cochlear spiral, represented in percentage from the apex. The number of inner hair cells was calculated for each 5% increment of the cochlear length.

The left cochlea was better dissected and exposed than the right. The cochlear length was 27.19 mm. In the left ear, because the basilar membrane was artefactually folded, the sensory cells of the organ of Corti from the hook region (88.6% to 99% from the apex) could not be assessed. However, the rest of the cochlea was well preserved, with some signs of post-mortem decomposition due to delay between the death of the individual and the fixation of the inner ear. The number of IHCs present and absent were counted every 5% of the cochlear length. There was little loss of IHCs in the apical region, up to 35% from the apex, and an increasing trend of IHC loss towards the base of up to 84.6% loss of IHCs at 80 to 85% of the apex (Figure 3). The exposed areas of the hook region featured a similar pattern of IHC loss. However, in the first 50 µm of the hook, IHCs were present, with the three first IHCs arranged in two rows (Figure 2e).

The right cochlea was well preserved, especially in the region of the apical and middle turns. However, there was a dissection and processing artifact that hampered the ultrastructural evaluation of the reticular lamina of the sensory epithelium in the majority of the basal turn. In those locations where the organ of Corti was visible, there was a comparable distribution of IHCs loss as in the left cochlea, while the OHCs appeared morphologically intact. However, as the regions of the base where the sensory cells were visible were limited, there was insufficient exposure of the right ear to confirm comparable severity in the bilateral loss of IHCs.

## 4. Discussion

Cochlear ultrastructural analysis of a neonate harbor seal showed an extremely rare pattern of selective loss of IHCs, which was particularly severe in the basal turn (Figure 2 and Figure 3). Since this individual was very young, probably around one month old, it is likely that the IHC loss was congenital.

In most cases of sensorineural hearing loss in terrestrial mammals, either due to noise exposure [25], ototoxic drugs exposure [26], or genetic anomalies, the loss of IHCs is associated with significant or complete loss of OHCs. Thus, OHCs tend to be the most vulnerable elements in the inner ear, and selective loss of IHCs is highly uncommon.

Conversely, systemic administration of the anti-neoplastic drug carboplatin in the chinchilla, damages IHCs, leaving the OHCs morphologically intact [27]. In other species (e.g., guinea pig), carboplatin causes loss of both IHCs and OHCs [28]. Review of medical records for this seal confirmed that no carboplatin or other ototoxic drugs were administrated.

Many cases of congenital hearing loss are due to viral infections during different stages of fetal development (see review by Karimi-Boroujeni and colleagues [29]). Although congenital cytomegalovirus is the leading non-genetic cause of sensorineural hearing loss in children [30,31,32], herpes simplex virus and rubella virus infections are also detected [29]. Loss of OHCs was described in an infant with congenital cytomegalovirus infection [33]. In addition, loss of IHCs and OHCs was observed in murine cytomegalovirus-infected mice, despite spiral ganglion cells and perilymphatic epithelial cells, but not hair cells, were sites of viral infection [34]. Schachtele and colleagues suggested that OHCs were more susceptible than IHCs to murine cytomegalovirus infection. Similarly, guinea pigs inoculated with herpes simplex virus showed a marked loss of OHCs, while changes in the IHCs were less apparent [35]. In humans, congenital rubella virus infection leads to bilateral sensorineural hearing loss through apoptosis in the stria vascularis and the organ of Corti [36], but both IHCs and OHCs were degenerated [37]. Cytomegalovirus and herpes simplex virus belong to the family *Herpesviridae*. Since this seal pup had lesions consistent with phocid herpesvirus infection, we considered the possibility that the ultrastructural features found in the inner ear of this individual were correlated with herpesvirus infection. However, selective IHC loss was not previously described with a congenital viral infection, making a viral etiology unlikely.

Selective IHC loss was reported in the Bronx waltzer mutant mice (bv/bv), where the IHCs were either absent or abnormally haired, but the OHCs appeared normal [38]. Selective IHC loss was also shown in a mutant mouse with targeted deletion of high-affinity thiamine transporter gene SLC19A2 [39]. However, the IHC loss was observed throughout the cochlear spiral in the Bronx waltzer mutant mice [38], or more severe in the upper basal than the apical turn [40], and starting in the apical turn of the cochlea in the thiamine Slc19a2-null mice [39], but not specifically or with higher severity in the base of the cochlea. The phenotypes resulting from the two mutations reported in mice are not consistent with the pathological pattern found in this harbor seal.

Connexin 26 mutation (encoded by the GJB2 gene) is considered the most common cause for non-syndromic hereditary deafness [41,42]. In this disorder, the OHCs were more vulnerable than IHCs [43]. Therefore, it is unlikely that the pathogenesis observed in our individual might be due to the mutation of Connexin 26, one of the most common birth defects in humans.

Auditory neuropathy spectrum disorder refers to several hearing dysfunctions characterized by compromised signal processing along the auditory nerve or by deficient transmission of this signal to the auditory nerve by the presynaptic IHCs with normal function of OHCs (see review by De Siati and colleagues [44]). There is a wide range of localization of anatomic sites of impaired function, ranging from the region of IHCs synapses to the auditory neural fibers [45,46]. “Auditory synaptopathy” is the term used for auditory neuropathy spectrum disorder due to a defective or poorly functioning IHCs ribbon synapse, and the term “auditory neuropathy” when they are due to the dysfunction of neural fibers [46,47]. Cases of selective IHC loss were also reported nine-fold higher in premature infants in comparison to full-term infants [48]. Amatuzzi and colleagues proposed that a common cause of non-genetic auditory neuropathy spectrum disorder (called “auditory neuropathy” by the authors) can be a selective loss of IHCs rather than primary damage to the cochlear nerve.

On initial presentation to the MMR, this harbor seal neonate was not deemed premature. In addition, since the dissection of the cochlea prepared for SEM was optimized to image the sensory cells of the organ of Corti, it was not possible to evaluate if there was a degeneration of type I afferent neurons, precluding evaluation for potential auditory neuropathy spectrum disorder.

In summary, based on the review of human and laboratory studies, there are no apparent precedents that may account for the pattern of IHC loss observed in the harbor seal of our study. However, it is possible that harbor seals have unique hereditary diseases, distinct to humans and reported rodent models.

Since the disposition of the first sensory cells in the extreme hook in mammals can be variable [49], the finding of the three first IHCs disposed of in two rows (Figure 2e) in this harbor seal is documented but possibly not relevant.

Losses of up to 70% of IHCs and 50% of cochlear neurons were associated with a moderate elevation of hearing threshold (by an average of 20 dB SPL) in Bronx waltzer mutant mice [40]. As a result, for sufficient threshold hearing, not all IHCs might be required, particularly if the OHCs are present and functional [40]. In addition, carboplatin-induced IHC loss (ranging from 40 to 80%) in chinchillas had little effect on thresholds in quiet surroundings, but thresholds increased significantly when tested in the presence of broadband or narrowband noise [50]. Consequently, IHC loss or dysfunction may play a significant role in hearing-in-noise independent of OHC integrity, and these deficits may be present even when thresholds in quiet are within normal limits. Despite the harbor seal had other severe pathologies, it is likely that the lesions in the organ of Corti would have also caused difficulties for its survival.

This study highlights the importance to have baseline knowledge on “natural” congenital malformation of the hearing apparatus of marine mammals to be able to further differentiate from potential damage caused by exposure to factors (including noise) that the individuals might encounter during their lifetime.

## 5. Conclusions

This is the first study to report a case of selective IHC loss in a neonate marine mammal, likely congenital.

## Data Availability

The original contributions presented in the study are included in the article. Further inquiries can be directed to the corresponding author.

## References

[1] Smith R., Bale J., White K. (2005). Sensorineural hearing loss in children. Lancet.

[2] Belcher R., Virgin F., Duis J., Wootten C. (2021). Genetic and non-genetic workup for pediatric congenital hearing loss. Front. Pediatr..

[3] Morton N.E. (1991). Genetic epidemiology of hearing impairment. Ann. N. Y. Acad. Sci. USA.

[4] Raymond M., Walker E., Dave I., Dedhia K. (2019). Genetic testing for congenital non-syndromic sensorineural hearing loss. Int. J. Pediatr. Otorhinolaryngol..

[5] Kalatzis V., Petit C. (1998). The fundamental and medical impacts of recent progress in research on hereditary hearing loss. Hum. Mol. Genet..

[6] Farooq R., Hussain K., Tariq M., Farooq A., Mustafa M. (2020). CRISPR/Cas9: Targeted genome editing for the treatment of hereditary hearing loss. J. Appl. Genet..

[7] Bredberg G., Ades H.W., Engström H. (1972). Scanning electron microscopy of the normal and pathologically altered organ of Corti. Acta Oto-Laryngol..

[8] Hu B.H., Guo W., Wang P.Y., Henderson D., Jiang S.C. (2000). Intense noise-induced apoptosis in hair cells of guinea pig cochleae. Acta Oto-Laryngol..

[9] Raphael Y., Altschuler R.A. (1991). Reorganization of cytoskeletal and junctional proteins during cochlear hair cell degeneration. Cell Motil. Cytoskelet..

[10] Morell M., Brownlow A., McGovern B., Raverty S.A., Shadwick R.E., André M. (2017). Implementation of a method to visualize noise-induced hearing loss in mass stranded cetaceans. Sci. Rep..

[11] Suzuki M., Kishimoto M., Hayama S.-I., Ohtaishi N., Nakane F. (1992). A case of cleft palate in a Kuril seal (*Phoca vitulina* stejnegeri), from Hokkaido, Japan. J. Wildl. Dis..

[12] McKnight C.A., Reynolds T.L., Haulena M., Delahunta A., Gulland F.M.D. (2005). Congenital hemicerebral anomaly in a stranded Pacific harbor seal (*Phoca vitulina* richardsi). J. Wildl. Dis..

[13] Dennison S.E., Forrest L.J., Fleetwood M.L., Gulland F.M.D. (2009). Concurrent occipital bone malformation and atlantoaxial subluxation in a neonatal harbor seal (*Phoca vitulina*). J. Zoo Wildl. Med..

[14] Dennison S.E., Boor M., Fauquier D., Van Bonn W., Greig D.J., Gulland F.M.D. (2011). Foramen ovale and ductus arteriosus patency in neonatal harbor seal (*Phoca vitulina*) pups in rehabilitation. Aquat. Mamm..

[15] Harris H.S., Facemire P., Greig D.J., Colegrove K.M., Ylitalo G.M., Yanagida G.K., Nutter F.B., Fleetwood M., Gulland F.M.D. (2011). Congenital neuroglial heterotopia in a neonatal harbor seal (*Phoca vitulina* richardsi ) with evidence of recent exposure to polycyclic aromatic hydrocarbons. J. Wildl. Dis..

[16] Leger J.A.S., Nilson E.M. (2014). Intestinal atresia in a harbor seal (*Phoca vitulina*) and a review of congenital conditions of the species. Aquat. Mamm..

[17] D’Agnese E.R., Lambourn D.M., Olson J.K., Huggins J.L., Raverty S., Garner M.M., Calambokidis J., Scott A.A., Jeffries S.J., Gaydos J.K. (2021). Congenital diseases in harbor seals (*Phoca vitulina* richardsii) from the Salish Sea. J. Wildl. Dis..

[18] Geraci J.R., Lounsbury V.J. (2005). Marine Mammals Ashore: A Field Guide for Strandings.

[19] Raverty S.A., Duignan P.J., Jepson P.D., Morell M., Dierauf L.A., Gulland F.M. (2018). Gross Necropsy and Specimen Collection Protocols (Chapter 13). CRC Handbook of Marine Mammal Medicine.

[20] Morell M., Lenoir M., Shadwick R.E., Jauniaux T., Dabin W., Begeman L., Ferreira M., Maestre I., Degollada E., Hernandez-Milian G. (2015). Ultrastructure of the Odontocete organ of Corti: Scanning and transmission electron microscopy. J. Comp. Neurol..

[21] Morell M., Raverty S.A., Mulsow J., Haulena M., Barret-Lennard L., Nordstrom C., Venail F., Shadwick R.E. (2020). Combining cochlear analysis and auditory evoked potentials in a beluga whale with high-frequency hearing loss. Front. Vet. Sci..

[22] Morell M., Ijsseldijk L.L., Piscitelli-Doshkov M., Ostertag S., Estrade V., Haulena M., Doshkov P., Bourien J., Raverty S.A., Siebert U. (2021). Cochlear apical morphology in toothed whales: Using the pairing hair cell—Deiters’ cell as a marker to detect lesions. Anat. Rec. Adv. Integr. Anat. Evol. Biol..

[23] Girdlestone C.D., Ng J., Kössl M., Caplot A., Shadwick R.E., Morell M. (2020). Correlating cochlear morphometrics from Parnell’s mustached bat (*Pteronotus parnellii*) with hearing. J. Assoc. Res. Otolaryngol..

[24] Gibson A.K., Raverty S., Lambourn D.M., Huggins J., Magargal S.L., Grigg M.E. (2011). Polyparasitism is associated with in-creased disease severity in Toxoplasma gondii-infected marine sentinel species. PLoS Negl. Trop. Dis..

[25] Bohne A.B., Harding G.W. (2000). Degeneration in the cochlea after noise damage: Primary versus secondary events. Am. J. Otol..

[26] Huizing E.H., De Groot J.C.M.J. (1987). Human cochlear pathology in aminoglycoside ototoxicity—A review. Acta Oto-Laryngologica.

[27] Takeno S., Harrison R.V., Mount R.J., Wake M., Harada Y. (1994). Induction of selective inner hair cell damage by carboplatin. Scanning Microsc..

[28] Saito T., Saito H., Saito K., Wakui S., Manabe Y., Tsuda G. (1989). Ototoxicity of carboplatin in guinea pigs. Auris Nasus Larynx.

[29] Karimi-Boroujeni M., Zahedi-Amiri A., Coombs K.M. (2021). Embryonic origins of virus-induced hearing loss: Overview of molecular etiology. Viruses.

[30] Ogawa H., Suzutani T., Baba Y., Koyano S., Nozawa N., Ishibashi K., Fujieda K., Inoue N., Omori K. (2007). Etiology of severe sensorineural hearing loss in children: Independent impact of congenital cytomegalovirus infection and GJB2 mutations. J. Infect. Dis..

[31] Grosse S.D., Ross D.S., Dollard S.C. (2008). Congenital cytomegalovirus (CMV) infection as a cause of permanent bilateral hearing loss: A quantitative assessment. J. Clin. Virol..

[32] Cheeran M., Lokensgard J.R., Schleiss M.R. (2009). Neuropathogenesis of congenital cytomegalovirus infection: Disease mechanisms and prospects for intervention. Clin. Microbiol. Rev..

[33] Tsuprun V., Keskin N., Schleiss M.R., Schachern P., Cureoglu S. (2019). Cytomegalovirus-induced pathology in human temporal bones with congenital and acquired infection. Am. J. Otolaryngol..

[34] Schachtele S.J., Mutnal M.B., Schleiss M.R., Lokensgard J.R. (2011). Cytomegalovirus-induced sensorineural hearing loss with persistent cochlear inflammation in neonatal mice. J. NeuroVirology.

[35] Nomura Y., Kurata T., Saito K. (1985). Cochlear changes after Herpes simplex virus infection. Acta Oto-Laryngol..

[36] Cohen B.E., Durstenfeld A., Roehm P.C. (2014). Viral causes of hearing loss: A review for hearing health professionals. Trends Hear..

[37] Hemenway W.G., Sando I., McChesney D. (1969). Temporal bone pathology following maternal rubella. Arch. Klin. Exp. Ohr. Nas. Kehlk. Heilk..

[38] Lenoir M., Pujol R. (1984). Age-related structural investigation of the Bronx waltzer mutant mouse cochlea: Scanning and transmission electron microscopy. Hear. Res..

[39] Liberman M.C., Tartaglini E., Fleming J.C., Neufeld E.J. (2006). Deletion of SLC19A2, the high affinity Thiamine transporter, causes selective inner hair cell loss and an auditory neuropathy phenotype. J. Assoc. Res. Otolaryngol..

[40] Schrott A., Stephan K., Spoendlin H. (1989). Hearing with selective inner hair cell loss. Hear. Res..

[41] Kelsell D.P., Dunlop J., Stevens H.P., Lench N., Liang J.N., Parry G., Mueller R.F., Leigh I.M. (1997). Connexin 26 mutations in hereditary non-syndromic sensorineural deafness. Nature.

[42] Zelante L., Gasparini P., Estivill X., Melchionda S., D’Agruma L., Govea N., Mila M., Monica M.D., Lutfi J., Shohat M. (1997). Connexin26 mutations associated with the most common form of non-syndromic neurosensory autosomal recessive deafness (DFNB1) in Mediterraneans. Hum. Mol. Genet..

[43] Chen S., Sun Y., Lin X., Kong W. (2014). Down regulated connexin26 at different postnatal stage displayed different types of cellular degeneration and formation of organ of Corti. Biochem. Biophys. Res. Commun..

[44] De Siati R.D., Rosenzweig F., Gersdor G., Gregoire A., Rombaux P., Deggouj N. (2020). Auditory neuropathy spectrum disorders: From diagnosis to treatment: Literature review and case reports. J. Clin. Med..

[45] Moser T., Starr A. (2016). Auditory neuropathy–neural and synaptic mechanisms. Nat. Rev. Neurol..

[46] Shearer A.E., Hansen M.R. (2019). Auditory synaptopathy, auditory neuropathy, and cochlear implantation. Laryngoscope Investig. Otolaryngol..

[47] Foerst A., Beutner D., Lang-Roth R., Huttenbrink K.B., von Wedel H., Walger M. (2006). Prevalence of auditory neuropathy/synaptopathy in a population of children with profound hearing loss. Int. J. Pediatr. Otorhinolaryngol..

[48] Amatuzzi M., Liberman M.C., Northrop C. (2011). Selective inner hair cell loss in prematurity: A temporal bone study of infants from a neonatal intensive care unit. J. Assoc. Res. Otolaryngol..

[49] Soons J.A.M., Ricci A.J., Steele C.R., Puria S. (2014). Cytoarchitecture of the mouse organ of Corti from base to apex, determined using in situ two-photon imaging. J. Assoc. Res. Otolaryngol..

[50] Lobarinas E., Salvi R., Ding D. (2015). Selective inner hair cell dysfunction in chinchillas impairs hearing-in-noise in the absence of outer hair cell loss. J. Assoc. Res. Otolaryngol..

